# Ultrasound-Assisted Curdlan Curing Reduces Water Loss of Rabbit Meat: Water Retention Performance, Myofibrillar Protein Structure, and Processing Adaptability

**DOI:** 10.3390/foods15101748

**Published:** 2026-05-15

**Authors:** Zhuohang Li, Jiamin Zhang, Bo Hou, Jing Liao

**Affiliations:** 1 College of Food and Biological Engineering, Chengdu University, Chengdu 610106, China; 2 Meat Processing Key Laboratory of Sichuan Province, Chengdu University, Chengdu 610106, China; 3 Sichuan Provincial Engineering Research Center of Meat Quality Improvement and Safety Control Technology, Chengdu University, Chengdu 610106, China

**Keywords:** ultrasound, curdlan, rabbit meat, water retention performance

## Abstract

Improving the water-holding capacity (WHC) during the processing of rabbit meat can effectively enhance the texture of the final product, but it remains a practical challenge. This study aims to develop an ultrasound-assisted curdlan curing strategy to reduce the water loss of rabbit meat during the processing. Herein, the water retention performance, myofibrillar protein (MP) structure, and processing adaptability of rabbit meat as affected by the ultrasound-assisted curdlan curing treatment were investigated. Compared with the control group, ultrasound-assisted curdlan treatment increased WHC by 14.0% and reduced cooking loss by 15.4%. Moreover, this combined treatment showed significantly higher WHC and lower cooking loss than curdlan or ultrasound treatment alone (*p* < 0.05). Moreover, the ultrasound-assisted curdlan curing resulted in higher ultraviolet absorption and fluorescence intensity of myofibrillar proteins (MPs) in rabbit meat, but the intensity of the main protein band observed in SDS-PAGE was lower. Furthermore, the rabbit meat treated with the ultrasound-assisted curdlan curing maintains the highest water content (75.2% for steaming, 74.7% for boiling, 74.4% for microwaving, 70.1% for roasting, and 71.8% for air-frying) under various thermal processing methods. Therefore, the ultrasound-assisted curdlan curing offers a feasible route to improve water retention in rabbit meat, providing an applicable basis for reducing water loss in meat production.

## 1. Introduction

Rabbit meat has gained increasing attention due to its nutritional characteristics of being rich in protein and having a low fat content. However, the processing of rabbit meat can easily lead to water loss, thereby affecting the textures (e.g., tenderness) of the final products. Accordingly, reducing water loss in the processing of rabbit meat is generally regarded as the primary goal for controlling its quality [1]. The conventional water retention strategies for meat products mainly rely on phosphates, which can improve water-binding and texture by increasing ionic strength and shifting the protein environment toward conditions that favor swelling and hydration [2]. However, this “classic” solution has faced growing pressure from clean-label positioning [3], prompting a shift toward more natural functional systems that can preserve yield and eating quality without relying on phosphate-heavy formulations [2,4]. Recently, food polysaccharides have been widely used as natural water retention agents to improve the texture of meat due to their strong water absorption capacity and ability to interact with MPs [4,5].

Curdlan is a neutral and food-compatible polysaccharide with strong water-binding ability, making it a practical candidate for improving water retention in meat matrices [6]. In most cases, curdlan swells when heated and participates in the formation of a more compact and continuous gel structure, thereby limiting the mobility of water and fat within the protein matrix and improving cooking yield [7]. For example, incorporating curdlan into sausages within a low dose (0.1%) was reported to reduce cooking loss and improve emulsion stability [8]. Another study has also shown that curdlan-containing formulations can reduce cooking loss and strengthen the gel structure, as curdlan can reinforce the thermally induced protein network and entrap water more effectively during heating [9]. Moreover, curdlan has also been applied as a functional ingredient to modify the textural properties of beef emulsified sausage [10]. Nevertheless, curdlan exerts its functions depending on the effective binding to the developing protein scaffold of the meat; incomplete integration or unfavorable phase behavior may yield localized water-holding reinforcement rather than uniformly compact, water-stable networks [5,7].

Ultrasound is increasingly positioned as a non-thermal intensification tool that can promote water retention and texture improvement through cavitation-driven structural and protein-related effects, although the direction and magnitude of benefits depend strongly on the parameters [11]. It has been increasingly explored as a practical processing aid to improve the water-holding capacity (WHC) of meat and meat products [12,13,14], because acoustic cavitation and microstreaming can intensify mass transfer, modify muscle microstructure, and modulate MPs toward stronger water–matrix interactions. A recent study has shown that an ultrasound-assisted strategy can reduce water losses in meat products: for example, ultrasonic-assisted soaking with water-holding agents lowered centrifugal loss and limited water migration during frozen storage of beef [15]. In addition, ultrasound-assisted braising improved WHC of rabbit meat during cooking, as reflected by decreased cooking loss and a higher proportion of immobilized water [16]. Specifically, ultrasound-assisted heating or pretreatment induces partial protein unfolding, resulting in denser microstructures that trap water more effectively [17]. These benefits are supported by broader reviews, which report enhanced marination uptake, emulsion stability, and moisture retention across various meat applications [12,18]. Nevertheless, excessive or prolonged ultrasound can introduce quality penalties such as accelerated lipid oxidation or structural over-disruption that ultimately weakens water binding, highlighting the need for careful parameter optimization and supportive co-ingredients [12,16,19].

Overall, curdlan exhibits desirable hydration capacity and water-holding potential, whereas ultrasound can also increase the water-holding capacity through promoting protein structural reorganization. Previous studies on ultrasound combined with curdlan treatment have mainly focused on gel-type systems, such as surimi or low-salt comminuted meat products [20,21], whereas its application in a whole-muscle sample, especially rabbit meat, remains unexplored. It was hypothesized that the combination of curdlan and ultrasound treatment could further reduce water loss in rabbit meat during processing. Therefore, this study aimed to evaluate the effects of ultrasound-assisted curdlan curing on the water-holding properties of rabbit meat and to further interpret the related changes in myofibrillar protein structure. In this context, the present work provides a more integrated evaluation of this combined treatment in rabbit meat, including water-holding properties, structural characterization, and processing adaptability under different cooking methods.

## 2. Materials and Methods

### 2.1. Samples and Reagents

Fresh longissimus dorsi muscles were collected from 24 commercially slaughtered male rabbits purchased from the same local supplier in Chengdu, China. The samples were obtained within 6 h postmortem, individually packaged, and immediately transported to the laboratory under refrigerated conditions. Curdlan was obtained from Jiangsu Yiming Biological Technology Co., Ltd. (Taixing, China). Sodium chloride, potassium chloride, magnesium chloride hexahydrate, and ethanol were purchased from Sinopharm Chemical Reagent Co., Ltd. (Shanghai, China). Potassium bromide, isoamyl acetate, hydrochloric acid, and sodium hydroxide were purchased from Shanghai Aladdin Biochemical Technology Co., Ltd. (Shanghai, China). EGTA, SDS, potassium dihydrogen phosphate, dipotassium hydrogen phosphate, and glutaraldehyde fixative were purchased from Beijing Solarbio Science & Technology Co., Ltd. (Beijing, China). The SDS–PAGE fast gel preparation kit, prestained protein marker, Coomassie Brilliant Blue fast staining/destaining kit, and SDS–PAGE sample loading buffer were purchased from Beyotime Biotechnology (Haimen, China).

### 2.2. Water Retention Performance Evaluation

In this study, the rabbit longissimus dorsi muscles were selected for the evaluation of the water retention performance of ultrasound-assisted curdlan curing and cut into same-sized rabbit filets (1.0 cm × 1.0 cm × 1.0 cm) before subjecting to different treatments, including control, curdlan, ultrasound, and curdlan/ultrasound. For the control group, the rabbit fillets were held for 30 min without any treatments. For the curdlan group, the rabbit fillets were immersed in a 0.5% (*m*/*v*) curdlan solution for 30 min. For the ultrasound groups, the rabbit fillets were vacuum-packed and then immersed in an ice water bath for ultrasonic processing (with a power of 200 W for 5 min), then immersed in water for 25 min. For the curdlan/ultrasound group, the rabbit fillets were first subjected to the above-mentioned ultrasound conditions and then immersed in 0.5% curdlan for 25 min. For the preparation of cooked samples, the treated rabbit fillets were vacuum-packed and heated in a thermostatic water bath at 85 °C until the core temperature reached 80 °C. The internal temperature was monitored using a digital thermometer. After cooking, the samples were cooled to room temperature, and the surface moisture was gently removed with filter paper before further analysis.

Color analysis. The color of the samples was analyzed using a handheld colorimeter (CR-400, Konica Minolta, Tokyo, Japan) under illuminant D65, with a 10° standard observer and an 8 mm aperture. Before measurement, the instrument was calibrated with a white standard plate. Three readings were taken at different positions on the sample surface, and the average values were recorded as CIE L*, a*, and b* [22]. The ΔE value was calculated from the L*, a*, and b* coordinates relative to the reference sample [23].

pH determination. The pH values of the samples were determined using a portable insertion pH meter equipped with a penetration electrode. The electrode was inserted directly into the muscle, and each measurement was taken at three different locations.

WHC determination. The WHC of the processed rabbit filets was determined by a centrifugation-based method. Briefly, the samples were weighed (W_0_) and placed in a 15 mL tube lined with pre-dried filter paper, then centrifuged at 2500× *g* for 20 min at 4 °C. After centrifugation, the samples were blotted and reweighed (W_1_). WHC was calculated from mass loss and expressed as the percentage of water retained under these conditions [24,25,26].

$$\mathrm{WHC}\;(\%)=\frac{W_{1}}{W_{0}}\times 100\%$$

Cooking loss determination. The cooking loss of the processed rabbit filets was measured by a bagged water-bath method. Briefly, the samples were weighed (W_0_) and sealed in a heat-stable polyethylene bag. Subsequently, the bag was placed in a constant-temperature water bath and heated until the center temperature reached 75 °C. After cooling, the samples were blotted and reweighed (W_1_). Cooking loss was calculated from the mass difference before and after heating [27,28].

$$\mathrm{Cooking}\;\mathrm{loss}\;(\%)=(\frac{W_{0}-W_{1}}{W_{0}})\times 100\%$$

Microstructure observation. The microstructure of the processed rabbit filets was observed by using a scanning electron microscope (SEM) (Sigma 300, Carl Zeiss Microscopy GmbH, Jena, Germany). Before observation, the samples were fixed in 2.5% glutaraldehyde at 4 °C for 24 h, dehydrated in graded ethanol (60–100%), and exchanged with isoamyl acetate. After these steps, the prepared samples were freeze-dried, gold-sputtered, and imaged under high vacuum at a low accelerating voltage (~3.0 kV) [29,30].

Hardness and shear force determination. The hardness and shear force of the processed rabbit filets were measured using a texture analyzer (TA.XT Plus, Stable Micro Systems Ltd., Godalming, Surrey, UK). Before hardness analysis, the samples were cut into uniform blocks (1.0 cm × 1.0 cm × 1.0 cm) and double-compressed with a 36 mm cylindrical probe (P/36R) to 50% strain (two cycles, 5 s interval; trigger 5 g; pre-test/test/post-test speeds 2.0/1.0/1.0 mm/s), and the hardness was recorded from the peak force of the first compression [31,32]. Shear force was determined according to a previously reported method with minor modifications [33]. Briefly, cooked rabbit meat samples were cut into cube-shaped pieces of approximately 1.0 cm × 1.0 cm × 1.0 cm and sheared using a V-shaped blade. The blade distance, test speed, head speed, and trigger force were set at 22.0 mm, 21.0 mm/s, 21.0 mm/s, and 5.6 N, respectively. The peak force obtained during shearing was normalized to the sample cross-sectional area and expressed as N/cm^2^.

Water mobility and spatial distribution analysis. The water mobility and spatial distribution in rabbit meat were evaluated using a low-field nuclear magnetic resonance (LF-NMR) analyzer (NMI20-040V-I; Suzhou Niumag Analytical Instrument Co., Ltd., Suzhou, China) operating at 22.4 MHz with a magnet strength of 0.5 T. The magnet temperature was maintained at 32 °C during measurements to support stability and reproducibility [34]. Samples were trimmed to uniform size, wrapped with parafilm to limit moisture loss, and gently blotted to remove surface water before testing. Transverse relaxation time (T2) was measured using the Carr–Purcell–Meiboom–Gill (CPMG) sequence with the following parameters: waiting time (TW) = 4000 ms, echo time (TE) = 0.200 ms, number of echoes (NECH) = 6000, and number of scans (NS) = 4–8. The T2 decay curves were fitted using a multi-exponential model to generate the corresponding T2 relaxation distributions. Based on the resolved relaxation peaks, three water populations were distinguished and designated as T2b, T21, and T22. According to their relaxation characteristics and in line with previous LF-NMR studies on muscle food systems [35,36], these components were interpreted as bound water, immobilized water, and free water, respectively. The relative proportion of each population was calculated from the integrated area of its corresponding peak and expressed as a percentage of the total T2 peak area. To visually corroborate the LF-NMR results, magnetic resonance imaging (MRI) was performed using a spin-echo (SE) sequence to obtain proton density-weighted images (FOV = 80 mm × 80 mm; slice thickness = 4.0 mm; TE/TR ≈ 18.1/500 ms). The pseudo-color images were used to qualitatively assess moisture distribution and homogeneity within the rabbit meat matrix [34].

### 2.3. Myofibrillar Protein Structure Analysis

The structural analysis of the MPs extracted from the processed rabbit filets was performed to elucidate the water retention mechanisms of the ultrasonic-assisted curdlan curing process. In brief, the processed rabbit meat samples were minced and then homogenized with ice-cold extraction buffer (containing 100 mM KCl, 2 mM MgCl_2_, 1 mM EGTA/EDTA, 10 mM K_2_HPO_4_), filtered through four layers of gauze, and centrifuged at 8000 r/min for 15 min. The collected pellet was washed with fresh buffer three times, then dispersed in 0.1 M NaCl to obtain MPs, which were stored at −20 °C until analysis.

Microstructure observation. The microstructure of the obtained MPs was observed by using an SEM. Before observation, the equal-concentration MP samples were freeze-dried, gold-coated, and observed by SEM (Sigma 300, Carl Zeiss Microscopy GmbH, Jena, Germany) at 10.0 kV [37].

Zeta-potential measurement. The zeta-potential of the obtained MPs was measured by a Malvern ZEN 3600 (Malvern Instruments, Malvern, UK). Before measurement, MPs were dispersed in 0.02 M phosphate buffer (pH 6.0) containing 0.6 M NaCl and adjusted to 9 mg/mL. After equilibration for 30 min, the obtained MPs were measured at room temperature [38,39].

Ultraviolet spectra scan. Ultraviolet absorption spectra were recorded using a UV–Vis spectrophotometer (UV-2600i, Shimadzu Corporation, Kyoto, Japan) to characterize the absorption features of the protein dispersions. Before analysis, protein dispersions from each treatment were diluted with the same buffer to a unified protein concentration.

Fluorescence spectra scan. The intrinsic fluorescence spectra were recorded using a fluorescence spectrophotometer. Before analysis, myofibrillar protein (MP) was dispersed in 0.02 M phosphate buffer (pH 6.0) containing 0.6 M NaCl and diluted to 9 mg/mL, allowing equilibration after standing for 30 min. The excitation wavelength was set at 295 nm to preferentially excite tryptophan residues, and emission spectra were collected from 300 to 400 nm at a scanning speed of 240 nm/min, with fluorescence intensity recorded at 5 nm intervals [40].

SDS-PAGE analysis. SDS-PAGE was carried out to compare the obtained MP band patterns [41,42,43]. In brief, MP samples were adjusted to the same protein concentration, mixed with 5× SDS loading buffer (4:1, *v*/*v*), heated at 100 °C for 5 min, and cooled on ice. Proteins were separated on a 4% stacking gel and 10% resolving gel, with equal loading per lane and a prestained marker (BeyoColor™, 6.5–270 kDa; Beyotime, P0071, Beyotime Biotechnology Co., Ltd., Haimen, China) used without heating. Gels were stained with Coomassie Brilliant Blue R-250, destained, and imaged for qualitative comparison of band distribution and intensity.

Protein secondary structure analysis. FTIR was used to evaluate MP secondary structure [27,44,45]. In brief, freeze-dried MPs were mixed with spectroscopic-grade KBr, ground, and pressed into pellets, and spectra were collected using FTIR spectroscopy (Vector 33, Bruker, Germany). Secondary structure of MPs was quantified from the amide I region (1700–1600 cm^−1^) using baseline correction and second-derivative/deconvolution-assisted curve fitting; α-helix, β-sheet, β-turn, and random coil contents were calculated from fitted peak areas.

### 2.4. Processing Adaptability Analysis

Five different cooking methods (steaming, boiling, microwaving, air-frying, and roasting) were compared, and all samples were cooked to a unified core temperature of 80 °C to ensure comparable doneness. Before cooking, the processed rabbit filets were trimmed to similar size and thickness, and core temperature was continuously monitored by inserting a calibrated thermocouple into the geometric center; heating was stopped immediately once 80 °C was reached, followed by a 2–3 min resting period before analyses.

Redness determination. The redness (a*) of the cooked rabbit meat was measured using a handheld colorimeter in the CIE Lab* system (CR-400, Konica Minolta Sensing, Inc., Osaka, Japan).

Water content measurement. The water content of the cooked rabbit meat was measured by a drying method. In brief, the homogenized samples (approximately 3–5 g) were placed in a pre-dried and weighed plate (m_0_). Then, the total mass of the plate and the sample (m_1_) was recorded. Finally, samples were dried at 105 °C until constant weight, cooled in a desiccator, and reweighed (m_2_). The water content was calculated according to the following equation:

$$\mathrm{Water}\;\mathrm{content}\;\left(\%\right)=\frac{m_{1}-m_{2}}{m_{1}-m_{0}}\times 100\%$$

Smell characteristic analysis. Smell characteristics of the samples were analyzed using a PEN3 electronic nose (Airsense Analytics GmbH, Schwerin, Germany) according to a previously reported method with minor modifications [46]. Briefly, rabbit meat samples (3.0 g) from each treatment were homogenized and then transferred into a 20 mL sealed headspace vial. The vials were equilibrated in a water bath at 40 °C for 30 min, after which the headspace was introduced into the sensor chamber for measurement.

Hardness and chewiness evaluation. Hardness and chewiness of cooked rabbit meat were determined by texture profile analysis (TPA) using a texture analyzer [47,48]. Briefly, cooked samples were equilibrated to a consistent serving temperature and cut into uniform pieces. A two-cycle compression test was conducted with a cylindrical probe at a fixed deformation level (commonly set as a percentage of the original sample height) and a constant crosshead speed, following widely used TPA procedures. Hardness was recorded as the maximum force during the first compression cycle, while chewiness was calculated as hardness × cohesiveness × springiness, as defined in standard TPA output. Each sample was measured in triplicate, and the mean value was used for statistical analysis.

### 2.5. Statistical Analysis

The data were analyzed in SPSS 26.0 software (IBM, Corp., Armonk, NY, USA) with one-way ANOVA (general linear model [GLM]). Duncan’s multiple comparison test was employed to detect significant mean differences among the different treatment groups. Each measurement (*n* = 6) was repeated three times, and the results were expressed as mean ± standard error (SE), with statistical significance set at *p* < 0.05.

## 3. Results and Discussion

### 3.1. Water Retention Performance

**Figure 1:**
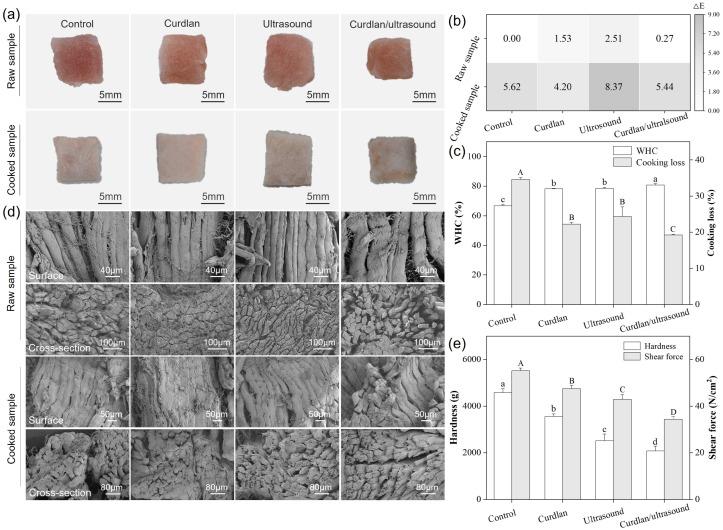
Optical images (**a**), ΔE values (**b**), WHC/cooking loss (**c**), microscopic morphology (**d**), and hardness/shear force (**e**) of different processed rabbit meat. Different capital letters and lowercase letters indicate significant differences (*p* < 0.05).

The appearance of rabbit meat subjected to different treatments is shown in Figure 1a. The raw samples exhibit a comparable red color without distinguishable visual differences among the control, curdlan, ultrasound, and curdlan/ultrasound treatments. While the cooked samples show a whitish color typical of cooked meat, these treatments did not result in abnormal darkening, excessive bleaching, or noticeable color changes. This indicates that neither curdlan addition nor ultrasound (alone or in combination) impaired the visual quality of rabbit meat. These visual observations were consistent with the ΔE values (Figure 1b), which also reflect the intensity of the color changes after cooking. These findings align with previous studies reporting that properly controlled ultrasound treatment does not adversely affect meat color [49,50,51], and curdlan incorporation maintains normal color attributes in meat products [52,53]. In this study, we also demonstrated that the ultrasound-assisted curdlan curing did not adversely affect meat appearance.

WHC and cooking loss are critical indicators reflecting the water retention performance of meat and meat products. In this study, these two parameters were measured for rabbit meat, and the results are shown in Figure 1c. The WHC of raw rabbit meat increased from 66.7% in the control to 78.2% in the curdlan group, 78.5% in the ultrasound group, and 80.7% in the curdlan/ultrasound group. Since pH is a crucial parameter that affects WHC, this study also measured the pH values of rabbit meat in each treatment group, which were 5.91, 5.86, 5.89, and 5.94 for control, curdlan, ultrasound, and curdlan/ultrasound, respectively. On the other hand, the cooking loss determination result (Figure 1c) shows that the rabbit meat in the control group exhibits a cooking loss of 34.6%, which is markedly higher than the rabbit meat in the curdlan group (22.2%), ultrasound group (24.4%), and curdlan/ultrasound group (19.2%). Curdlan has been shown to have the ability to enhance water retention performance in meat products by strengthening the gel matrix and reducing thermally induced fluid release [9,52]. In addition, ultrasound treatment has also been widely reported to increase WHC by modifying muscle microstructure and enhancing water immobilization [50,54,55]. Additionally, the ultrasound-assisted cooking processes have likewise been shown to reduce cooking loss and improve water retention in pork meatballs and spiced beef relative to conventional heating [56,57,58]. Overall, our results demonstrate that both curdlan and ultrasound treatment can improve the water retention performance of rabbit meat, as reflected by increased WHC and reduced cooking loss, while the combined treatment is more effective in improving overall water retention during cooking than either single treatment.

To validate the improvement of water retention performance in the rabbit meat, the microstructure of the processed rabbit meat was observed using an SEM. The SEM observation results provided supportive morphological evidence and may help explain the observed differences in WHC among treatments. As illustrated in Figure 1d, muscle fibers of the rabbit meat in the control group are tightly packed, with an intact myofibrillar network and only narrow endomysial and perimysial spaces, indicating limited room for water to be entrapped. In contrast, the rabbit meat treated with curdlan and ultrasound alone shows a slightly looser myofibrillar arrangement, small gaps emerge between adjacent fiber bundles, and the connective tissue matrix becomes less compact. The samples in the curdlan/ultrasound group exhibit the loosest and most porous structure, characterized by pronounced separation among fibers, swollen connective tissue regions, and a highly open fiber network in both cross-sectional and surface views, in line with the highest WHC and lowest cooking loss observed for this treatment. Similar SEM features, such as greater fiber separation, increased myofibrillar swelling, and thickened or disrupted perimysium, have been described in the ultrasound-assisted brined or marinated pork, where cavitation-induced mechanical effects promote microstructural loosening and are associated with improved water-holding and texture [12,59,60]. These observations are also consistent with classical structural models of water-holding capacity, which propose that water is mainly located in intra- and extra-myofibrillar spaces and that changes in myofibrillar spacing and fiber-bundle geometry critically determine the capacity of meat to retain water during processing [61]. Consequently, the enlarged interfiber and perimysial spaces observed in the combined curdlan and ultrasound treatment group may provide more space for immobilized water, which could help explain the higher WHC of this treatment compared with the other groups.

**Figure 2:**
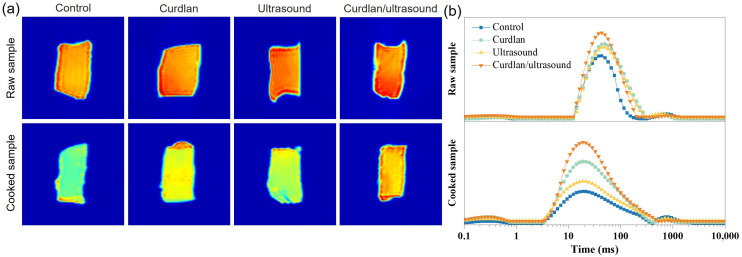
Pseudo-color proton-density MRI images (**a**) and T_2_ relaxation time distribution curves (**b**) of different processed rabbit meat.

The enhanced water retention performance of meat products contributes to improving their tenderness and texture. As a result, the texture parameters, including the hardness and shear force, of the rabbit meat were assessed. As depicted in Figure 1e, the rabbit meat in the control group exhibits the highest hardness (4591.5 g) and shear force (55.2 N/cm^2^), indicative of a relatively firm and tough texture. While the rabbit meat treated with curdlan and ultrasound shows a reduction in both parameters, with values of (3562.4 g and 47.5 N/cm^2^) and (2507.2 g and 42.9 N/cm^2^), respectively. It should be noted that the rabbit meat treated with curdlan/ultrasound shows the lowest values, with a hardness of 2073.8 g and a shear force of 34.4 N/cm^2^. Our results demonstrate that the ultrasound-assisted curdlan treatment showed the greatest improvement in tenderness among all treatments. This phenomenon of improving tenderness is consistent with the previous work on ultrasound-assisted tenderization of pork and duck meat, where ultrasound pretreatment or ultrasound-assisted marination significantly lowered shear force and increased sensory tenderness scores compared with conventionally processed controls [12,19,62,63]. Ultrasound-induced cavitation has been shown to promote myofibrillar fragmentation, disrupt Z-lines and partially loosen perimysial and endomysial connective tissue, thereby weakening fiber–fiber interactions and reducing resistance to compression and shearing [62,64]. In this study, curdlan treatment produced intermediate hardness and shear force values of rabbit meat, which agrees with its ability to form a three-dimensional β-(1→3)-glucan gel network within meat systems. Studies on pork myofibrillar and myosin model gels have shown that moderate curdlan levels (around 1.0%) increase WHC, gel strength, and storage modulus while generating a more uniform, compact network structure, although hardness may increase slightly depending on the concentration and matrix [52,65]. In low-fat or comminuted meat products, curdlan-containing formulations generally exhibit reduced cooking loss and improved juiciness, which can alleviate perceived toughness despite these changes in gel firmness [6,52]. Overall, the WHC, microstructures, and texture results in the present study suggest that the curdlan–ultrasound treatment optimized the balance between structural loosening and gel network formation, yielding cooked rabbit meat with a more open microstructure and markedly improved instrumental tenderness. In this study, the water distribution of these processed rabbit meats was also evaluated by LF-NMR. Figure 2a shows the pseudo-color proton-density MRI images illustrating the spatial water distribution in raw and cooked rabbit meat for each treatment. The color intensity in these images correlates with local hydrogen proton density (water content), where warmer colors (red/yellow) indicate regions of higher water content and cooler blue tones denote lower moisture [66]. In the raw samples, all groups exhibit relatively uniform internal water distribution, but the curdlan/ultrasound-treated meat already shows a subtly higher overall signal intensity (more extensive warm-colored areas) compared to the control, suggesting enhanced initial hydration or water binding. Upon cooking, pronounced differences emerge: the control and single-treatment samples (curdlan alone or ultrasound alone) display a notable reduction in internal moisture signal–evident as expanded blue zones, consistent with moisture migration and drip loss [67]. In contrast, the curdlan/ultrasound treatment retains a strong proton signal throughout the tissue (predominantly red/yellow in the core), indicating superior water retention even after cooking. Notably, in both the raw and cooked states, the combined curdlan and ultrasound treatment showed the highest water content and a more homogeneous water distribution, indicating its effectiveness in retaining water within the muscle matrix. These visual observations align with reports that ultrasound-assisted protein/polysaccharide treatments produce deeper and more widespread red intensity in MRI moisture maps, signifying denser networks that hold water more effectively [66]. The combined treatment likely reinforces the microstructure via curdlan’s gelation and ultrasound-induced protein modifications, thereby minimizing water migration and preserving internal moisture.

The LF-NMR T2 relaxation spectra of the samples is displayed in Figure 2b, which reflects the distribution of water populations with different mobilities. In general, the T21 relaxation time (10–100 ms) indicates immobilized water within the myofibrillar protein network [68]. In raw rabbit meat, the control group, curdlan group, ultrasound group, and curdlan/ultrasound group exhibit T21 relaxation times of 43.29 ms, 49.77 ms, 49.77 ms, and 43.29 ms. In cooked rabbit meat, these groups show the T21 relaxation times of 18.74 ms, 18.74 ms, 18.74 ms, and 18.74 ms. On the other hand, the corresponding relative peak-area proportions can be expressed as P21 [69]. As shown in Figure 2b, compared with the control group, the combined curdlan/ultrasound treatment increased P21 from 93.19% to 97.52% in raw rabbit meat and from 87.94% to 95.93% in cooked rabbit meat. These results indicate that the improvement in water-holding capacity was mainly associated with an increase in the immobilized-water fraction. In other words, the combined treatment promoted the retention of water in a more restricted and entrapped state, with this effect becoming more pronounced after cooking. This interpretation is consistent with previous LF-NMR studies showing that a higher proportion of immobilized water is generally associated with better water retention in meat products [70,71].

### 3.2. Myofibrillar Protein Structure

**Figure 3:**
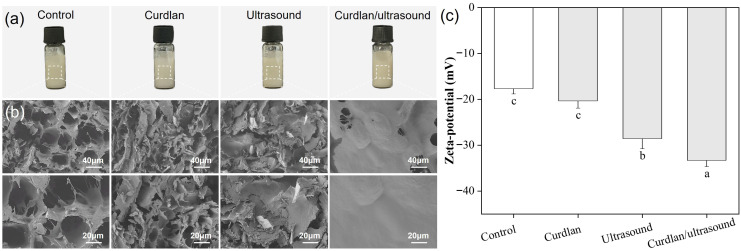
Optical images (**a**), SEM images (**b**), and zeta-potential (**c**) of MPs extracted from different processed rabbit meat. Different lowercase letters indicate significant differences (*p* < 0.05).

The water retention mechanism of ultrasound-assisted curdlan curing on rabbit meat was evaluated by monitoring structure changes in MPs in this study. Figure 3a illustrates the optical images of the extracted MPs, with no discernible precipitation/flocculation or phase separation among treatments. SEM images (Figure 3b) of the freeze-dried MPs show distinct treatment-related differences in the compactness and continuity of the solid matrix. The control group displayed a loose and discontinuous morphology with large interstitial gaps and abundant open voids, giving an obviously porous appearance after lyophilization. The curdlan group appeared heterogeneous, with intertwined flocculent domains and irregular aggregated regions embedded within the matrix, resulting in a rough and non-uniform surface. The ultrasound group looked comparatively scattered and disordered, with fragmented domains and uneven pore distribution, indicating that ultrasound alone did not yield a coherent continuous MP matrix under the current conditions. In contrast, the curdlan/ultrasound group exhibited the smoothest and most integrated morphology, characterized by a more continuous surface and markedly reduced pore formation, suggesting a denser MP-based solid matrix with fewer structural discontinuities. Freeze-drying removes ice by sublimation, and the resulting “pore/void” structure is strongly governed by the ice-crystal morphology and by the ability of the surrounding solid matrix to resist collapse; therefore, materials with weaker or more heterogeneous matrices typically show larger cavities and more open channels, while better-structured matrices can exhibit smaller pores and a more uniform appearance [72]. Curdlan is widely used for its viscosifying and water-holding functionality, and in protein–polysaccharide systems it can promote physical entanglement and hydrogen-bonding interactions but may also generate local aggregation or phase heterogeneity if dispersion is insufficient [6,73]. High-intensity ultrasound can modulate MP by cavitation-driven shear and microstreaming, typically decreasing particle size/turbidity and altering protein conformation and aggregation pathways, yet the final structural outcome is highly condition-dependent and may appear disordered when fragmentation and re-aggregation are not balanced [74,75]. Consequently, the particularly smooth and low-porosity morphology of the curdlan/ultrasound group can be reasonably attributed to improved MP–curdlan dispersion and a more homogeneous pre-freezing matrix that limits the development of large ice-templated voids during lyophilization, ultimately yielding a more continuous solid architecture after sublimation [6,72,74].

**Figure 4:**
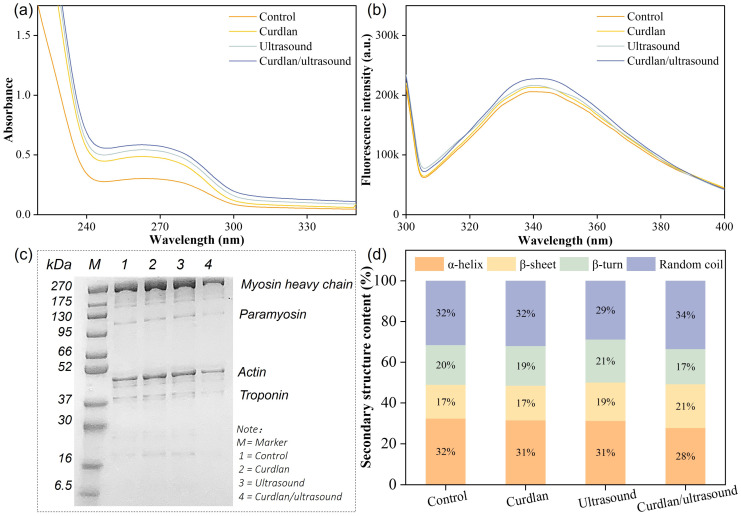
Ultraviolet spectra (**a**), fluorescence spectra (**b**), SDS–PAGE profiles (**c**), and secondary structure content (**d**) of MPs extracted from different processed rabbit meat.

The zeta-potential of the extracted MPs was determined, and the results are shown in Figure 3b. All MPs exhibited negative zeta-potential values (from −17.6 to −33.3 mV), indicating that the dispersed MPs carried net negative surface charges under the present measurement conditions. The negative sign is expected for MP suspensions when the measurement pH is above (or sufficiently away from) the isoelectric region of MPs, because deprotonation of ionizable amino-acid side chains (e.g., carboxyl groups) yields an excess of negative charges at the particle surface; accordingly, porcine MP suspensions have been reported to remain negatively charged across pH 4.9–7.0 [76], and “all MP suspensions exhibited a net negative charge” at neutral pH due to deprotonation [77]. Specifically, the control group showed a zeta-potential of −17.6 mV, while the curdlan group, ultrasound group, and curdlan/ultrasound group exhibited zeta-potentials of −20.3 mV, −28.5 mV, and −33.3 mV, respectively. Typically, high-intensity ultrasound can dissociate filamentous myosin assemblies and partially unfold MP, thereby exposing additional charged residues and strengthening electrostatic repulsion, which improves aqueous dispersion stability and inhibits re-association/aggregation [77,78]. On the other hand, electrostatic charge is a central driver of myofibrillar swelling and water association in muscle systems: shifting protein net charge away from the isoelectric point increases repulsion within the myofibrillar lattice and is linked to greater water retention [79]. The ultraviolet spectra of rabbit meat MPs are shown in Figure 4a. All treatments retained a comparable spectral profile (steep deep-UV decay and a broad near-UV hump across 240–300 nm), which is typical for MPs where aromatic residues contribute strongly in the 270–285 nm region and the overall band envelope is sensitive to the tertiary microenvironment rather than the appearance of new chromophores [80]. The increase in absorbance intensity (curdlan/ultrasound > ultrasound > curdlan > control), especially across the near-UV shoulder/plateau, is most consistent with greater solvent exposure of aromatic side chains and a looser tertiary packing state after treatment, an interpretation widely used for MP when absorbance near 280 nm rises following physical modification [81]. Generally, ultrasound cavitation can disrupt noncovalent constraints within the myosin filament assembly, promoting partial unfolding and reorganization that increases residue exposure under moderate conditions, while still preserving the overall aromatic signature [82]. This type of controllable conformational “opening” is functionally important because it can increase hydration sites and facilitate intermolecular associations during subsequent structuring, rather than merely raising optical density. The highest UV absorbance observed in the combined curdlan and ultrasound treatment suggests more pronounced changes in myofibrillar protein structure, which may have favored protein–polysaccharide interactions and contributed to the improved water-holding performance of this group [67].

The intrinsic fluorescence spectra of rabbit meat MPs exhibited a main emission band with a maximum emission wavelength (λmax) at 342 nm (Figure 4b), which is typical for tryptophan-dominated intrinsic fluorescence and is widely used to track changes in protein tertiary packing and local quenching environments [83,84,85,86]. Compared with the control, no obvious shift in λmax was observed in the curdlan-treated sample, whereas a slight red-shift appeared after ultrasound treatment and became more evident in the curdlan/ultrasound group. This result suggests that some aromatic residues were exposed to a relatively more polar microenvironment, reflecting conformational rearrangement of myofibrillar proteins [84,85,86]. In addition, the fluorescence intensity increased in the order of curdlan/ultrasound > ultrasound > curdlan > control, indicating that the combined treatment induced a more pronounced alteration in the tertiary structure of MPs. The relatively small change in λmax suggests that the polarity around the dominant Trp residues was only moderately affected, whereas the increased fluorescence intensity may be related to treatment-induced conformational rearrangement and altered local interactions around Trp residues [83,85]. Overall, the fluorescence results further support that curdlan/ultrasound treatment caused greater modification of the tertiary structure of MPs, which was in agreement with its improved water-holding capacity.

The SDS–PAGE profiles of MPs extracted from rabbit meat in the control, curdlan, ultrasound, and curdlan/ultrasound groups exhibited a largely conserved banding pattern (Figure 4c), indicating that the major contractile-protein repertoire was retained. It has been observed that a dominant band in the high–molecular-weight region is consistent with the myosin heavy chain (MHC, ~200 kDa) [87]; a band located between 95 and 130 kDa is the paramyosin [88]; an intense band at ~42 kDa corresponds to actin [89]; and bands in the ~30–40 kDa range are consistent with troponin subunits [90]. Importantly, no conspicuous enrichment of low–molecular-weight fragments was observed across treatments, which argues against extensive proteolytic breakdown as the dominant outcome of curdlan addition or ultrasound exposure in this study. It is worth noting that a notable qualitative feature of the gel is the overall paler staining of the curdlan/ultrasound lane relative to the other groups. When ultrasound is combined with curdlan, improved gelation and WHC have been reported in muscle protein systems, consistent with a shift in proteins from a readily soluble state toward a more integrated, water-immobilizing matrix [91]. Therefore, the SDS–PAGE result indicates that the superior water-holding performance of the curdlan/ultrasound treatment is driven primarily by treatment-induced reorganization and strengthened association of MPs into a hydrated network, rather than by extensive proteolysis.

The secondary structure content was determined via Amide I deconvolution. As shown in Figure 4d, rabbit meat MPs retained a broadly balanced secondary-structure profile in the control, with α-helix and random coil each accounting for 32%, accompanied by 17% β-sheet and 20% β-turn. Curdlan alone produced only marginal shifts (α-helix 31%, β-sheet 17%, β-turn 19%, random coil 32%), suggesting limited MP conformational reorganization under the current formulation. Ultrasound alone increased β-sheet (to 19%) and β-turn (to 21%) while reducing random coil (to 29%), consistent with ultrasound-driven cavitation and microstreaming that can disrupt intramolecular packing and re-route refolding/association toward β-structure enrichment under some conditions [75]. Another study also reported for ultrasound-treated fish MPs where β-sheet/random-coil populations were altered alongside other physicochemical changes [92]. Notably, the curdlan/ultrasound treatment displayed the lowest α-helix fraction (28%) and the highest β-sheet fraction (21%), together with an elevated random coil fraction (34%) and reduced β-turn (17%). This implies a mixed outcome of enhanced unfolding/helix loss yet incomplete ordering into β-turn, plausibly due to polysaccharide-mediated steric constraints and altered hydration that stabilize disordered segments while still promoting β-sheet-associated intermolecular hydrogen bonding [93,94,95,96].

### 3.3. Processing Adaptability

**Figure 5:**
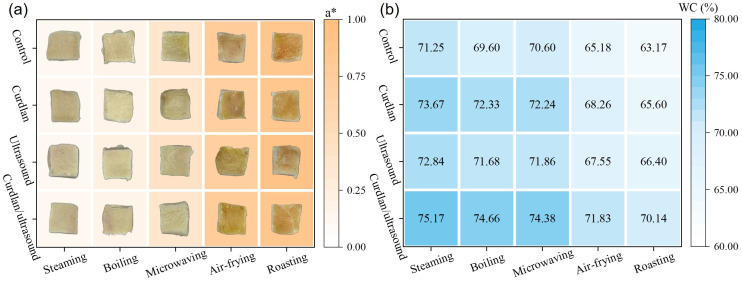
Redness value (**a**), and water content (**b**) of rabbit meat under different cooking treatments.

The appearance and redness values of rabbit meat after different cooking processing treatments are shown in Figure 5a. It has been found that the cooked rabbit meat surfaces showed a pronounced method-driven shift in hue, from a pale, low-browning appearance under wet-heat processing (steaming and boiling) to progressively darker, more golden–brown surfaces after microwaving, and the strongest browning after air-frying and roasting. These observations are in line with established meat-color frameworks from the instrumental a* values. Normalized a* remained low after steaming (0.12–0.15) and boiling (0.16–0.20), increased to intermediate values after microwaving (0.36–0.40), then rose sharply under air-frying (0.71–0.79) and reached the highest levels under roasting (0.86–0.92). This stepwise increase is compatible with the stronger dehydration and higher effective surface temperatures typical of dry-heat processing, which accelerate non-enzymatic browning chemistry (Maillard-type pathways, often coupled with lipid-derived reactions) and thereby deepen the red/brown component of the surface color [97,98]. Also, we found that among all the cooking methods, the differences in a* values between different curing methods were relatively small. Therefore, the ultrasound-assisted curdlan curing used in this study does not affect the appearance of the processed rabbit meat.

The water content of rabbit meat after different cooking processing treatments is shown in Figure 5b. Generally, the water content of meat products depends strongly on the processing method, with dry-heat conditions producing the lowest values overall, consistent with heat-induced protein denaturation/shrinkage and enhanced evaporative loss that jointly weaken water retention in cooked meat products [99,100]. In this study, the curdlan/ultrasound group consistently exhibited the highest water content under all cooking methods, with values of 75.2% for steaming, 74.7% for boiling, 74.4% for microwaving, 70.1% for roasting, and 71.8% for air-frying. These values were significantly higher than those of the other treatment groups (*p* < 0.05). This finding further supports the positive effect of the combined treatment on water retention during different cooking procedures.

**Figure 6:**
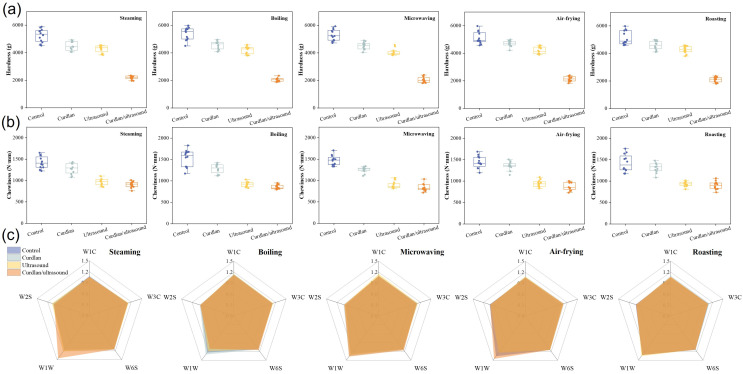
Hardness (**a**), chewiness (**b**), and smell characteristics (**c**) of rabbit meat under different cooking treatments.

The hardness of the rabbit meat exhibited a highly coherent treatment signature under steaming, boiling, microwaving, air-frying, and roasting, with the control samples consistently occupying the highest range (from 4.5 to 6.0 × 10^3^ g), curdlan and ultrasound clustering in the middle range (from 3.8 to 5.0 × 10^3^ g), and curdlan/ultrasound in the low hardness range (from 1.8 to 2.4 × 10^3^ g) (Figure 6a). Ultrasonic treatment has been found to lead to a decrease in the mechanical hardness of cooked poultry meat, and this phenomenon can be attributed to the mechanism of microstructure disruption [11,14]. Additionally, curdlan can modulate protein network organization and water retention in meat products, thereby exerting subsequent effects on the texture [9,65,101]. Our results suggest that rabbit meat treated with curdlan/ultrasound has the lowest hardness regardless of the cooking method used, which is beneficial for improving the texture of meat products during heat processing.

The chewiness of the rabbit meat also shifted downward across all five cooking methods, with control consistently exhibiting the highest chewiness (from 1.25 to 1.75 × 10^3^ N·mm), curdlan remaining intermediate (from 1.10 to 1.50 × 10^3^ N·mm), and both ultrasound and curdlan/ultrasound clustering in a lower band (from 0.75 to 1.05 × 10^3^ N·mm) (Figure 6b). Chewiness in texture profile analysis represents the mechanical work required to masticate a sample and is derived from hardness together with cohesiveness and springiness [102]. The ultrasound-associated chewiness reduction has been reported in poultry meat [14]. It has been shown that high-intensity ultrasound is a promising non-thermal route to attenuate meat toughness under optimized conditions [64]. The parallel downward movement of hardness and chewiness—most evident for ultrasound and curdlan/ultrasound—supports an overall tenderizing effect in cooked rabbit meat.

The electronic-nose radar charts show substantial superposition among the control, curdlan, ultrasound, and curdlan/ultrasound groups under steaming, boiling, microwaving, air-frying, and roasting (Figure 6c). This indicates that the processing methods used in this study did not produce marked differences in odor-related sensor response patterns after cooking. In general, thermal processing is likely to dominate volatile generation and release, whereas pretreatments may primarily affect the relative abundance of existing odor-active compounds rather than promote the formation of new odor classes [103]. Overall, the electronic-nose results suggest that the curdlan/ultrasound treatment did not introduce obvious deviations in volatile-response profiles across the different cooking methods.

Despite the ultrasound-assisted curdlan curing has exhibited good processing adaptability in this study, some limitations should be acknowledged when considering the practical application. First, curdlan has shown potential to improve the water-holding and textural properties of meat products, its performance is not necessarily consistent across different meat matrices and may be influenced by formulation composition and processing conditions [10]. As a result, the optimal addition level and practical compatibility of curdlan still need to be further verified in different rabbit meat. In addition, the present work was carried out under laboratory-scale conditions and focused mainly on changes in water-holding properties, texture, and myofibrillar protein characteristics of rabbit meat after ultrasound-assisted curdlan curing, its applicability in industrial processing remains to be confirmed. In particular, important factors related to commercial implementation, including process scale-up, equipment investment, production efficiency, and the additional cost associated with curdlan incorporation, were not evaluated in the present study. Further research is therefore needed to assess the feasibility, processing stability, and economic value of this treatment under pilot-scale and commercial production conditions.

## 4. Conclusions

In conclusion, this study demonstrates that ultrasound-assisted curdlan curing is an effective approach to reduce water loss of rabbit meat during processing. Compared with the curdlan and ultrasound alone, the ultrasound-assisted curdlan curing produces a more pronounced water retention performance. Moreover, this improvement has been proven to be related to the conformational remodeling and secondary-structure reorganization of MP, which promotes the reassembly of MP into a more compact gel network, thereby enhancing the water retention effect. Importantly, the ultrasound-assisted curdlan curing has shown excellent water retention ability when applied to different cooking treatments of rabbit meat, demonstrating good processing adaptability across practical cooking/thermal conditions. These findings support ultrasound-assisted curdlan curing as an effective processing strategy to reduce water losses and improve quality in rabbit meat products.

## Data Availability

The original contributions presented in this study are included in the article. Further inquiries can be directed to the corresponding authors.
